# Supplementary material for: Cancer-Specific miRNAs Extracted from Tissue-Exudative Extracellular Vesicles in Ovarian Clear Cell Carcinoma
Source: Int J Mol Sci. 2022 Dec 11;23(24):15715. doi: 10.3390/ijms232415715 (PMC9778693; doi:10.3390/ijms232415715)
Supplement: Supplementary file 1 [file ijms-23-15715-s001.zip › ijms-1962956-supplementary.pdf]

Supplemental Table S1. Te-EVs and RNA concentration in Tissue-exudative samples.

Sample	Protein concentrations of isolated Te-EVs (ng/nL)	RNA concentrations extracted from isolated TE-EVs (ng/μL)
Case 1 Cancer region	11.1	27.9
Case 1 Normal region	14.3	42.3
Case 2 Cancer region	13.4	31.7
Case 2 Normal region	8.6	23.4
Case 3 Cancer region	7.1	695.8
Case 3 Normal region	5.0	244.3
Case 4 Cancer region	6.3	88.9
Case 4 Normal region	6.9	67.7

Te-EVs, tissue-exudative extracellular vesicles;

Supplemental Table S2. Serum EVs and RNA concentrations in Blood samples.

Sample	Protein concentrations of isolated serum EVs (ng/nL)	RNA concentrations extracted from isolated serum EVs (ng/μL)
OCC Case 1 Before Surgery	3.3	4.1
OCC Case 1 After Surgery	4.8	2.1
OCC Case 2 Before Surgery	3.6	2.5
OCC Case 2 After Surgery	4.2	2.5
OCC Case 3 Before Surgery	9.3	4.0
OCC Case 3 After Surgery	3.8	2.8
OCC Case 4 Before Surgery	4.1	2.9
OCC Case 4 After Surgery	2.9	2.0
OCC Case 5 Before Surgery	3.6	2.1
OCC Case 5 After Surgery	3.8	2.1
OCC Case 6 Before Surgery	6.5	2.2
OCC Case 6 After Surgery	4.3	2.7
AEH Case 1 Before Surgery	4.5	2.8
AEH Case 1 After Surgery	4.1	4.7
AEH Case 2 Before Surgery	2.8	2.0
AEH Case 2 After Surgery	4.6	4.2
AEH Case 3 Before Surgery	5.8	2.5
AEH Case 3 After Surgery	4.6	2.1
AEH Case 4 Before Surgery	4.5	2.5
AEH Case 4 After Surgery	3.9	2.6

EV, Extracellular Vesicle; OCC, Ovarian Clear Cell carcinomas; AEH, Atypical Endometrial Hyperplasia;

Supplemental Table S3. Thirty-seven miRNAs were predominantly found in resected tissues of clear cell ovarian cancer.

ID	Probe Set Name	Transcript ID (Array Design)	Log Ratio
20501036	MIMAT0000617_st	hsa-miR-200c-3p	8.469065
20501163	MIMAT0000682_st	hsa-miR-200a-3p	7.118238
20500556	MIMAT0000318_st	hsa-miR-200b-3p	7.099837
20500450	MIMAT0000259_st	hsa-miR-182-5p	6.108348
20500459	MIMAT0000264_st	hsa-miR-203a	5.970606
20500555	MIMAT0004571_st	hsa-miR-200b-5p	5.915134
20500452	MIMAT0000261_st	hsa-miR-183-5p	4.870087
20500748	MIMAT0000432_st	hsa-miR-141-3p	4.775663
20504572	MIMAT0005797_st	hsa-miR-1301-3p	4.716464
20504433	MIMAT0003339_st	hsa-miR-421	4.22219
20500162	MIMAT0000087_st	hsa-miR-30a-5p	4.074956
20500163	MIMAT0000088_st	hsa-miR-30a-3p	4.027858
20500141	MIMAT0000076_st	hsa-miR-21-5p	3.845076
20501162	MIMAT0001620_st	hsa-miR-200a-5p	3.820005
20500465	MIMAT0000267_st	hsa-miR-210-3p	3.353928
20504408	MIMAT0003322_st	hsa-miR-652-3p	3.207805
20501276	MIMAT0000751_st	hsa-miR-330-3p	3.02951
20518788	MIMAT0018932_st	hsa-miR-378f	2.74446
20502129	MIMAT0003393_st	hsa-miR-425-5p	2.737563
20501312	MIMAT0000772_st	hsa-miR-345-5p	2.62025
20501181	MIMAT0000691_st	hsa-miR-130b-3p	2.364236
20500453	MIMAT0004560_st	hsa-miR-183-3p	2.299574
20500173	MIMAT0000093_st	hsa-miR-93-5p	2.07324
20500196	MIMAT0000104_st	hsa-miR-107	1.979804
20518834	MIMAT0018976_st	hsa-miR-4454	1.933004
20500718	MIMAT0000417_st	hsa-miR-15b-5p	1.916491
20517675	MIMAT0016847_st	hsa-miR-378c	1.906211
20500423	MIMAT0004550_st	hsa-miR-30c-2-3p	1.903362
20500191	MIMAT0000101_st	hsa-miR-103a-3p	1.84253
20525434	MIMAT0027373_st	hsa-miR-6736-5p	1.842042
20501237	MIMAT0000728_st	hsa-miR-375	1.842041
20518925	MIMAT0019064_st	hsa-miR-4525	1.468871
20500418	MIMAT0000242_st	hsa-miR-129-5p	1.337035
20509229	MIMAT0026917_st	hsa-miR-1910-3p	1.202884
20518843	MIMAT0018985_st	hsa-miR-3135b	1.201568
20500194	MIMAT0000103_st	hsa-miR-106a-5p	1.041396
20500130	MIMAT0000070_st	hsa-miR-17-5p	1.041211

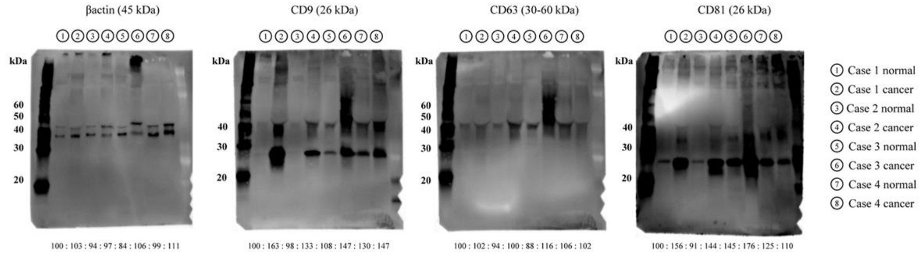

Figure S1. Western blot analysis was performed to all Te-EVs obtained from cancer and normal specimens in each case.
